# Mothers and Babies Online Course: Participant Characteristics and Behaviors in a Web-Based Prevention of Postpartum Depression Intervention

**DOI:** 10.3389/fgwh.2022.846611

**Published:** 2022-06-24

**Authors:** Alinne Z. Barrera, Sydney Y. Morris, Adriana Ruiz

**Affiliations:** Department of Psychology, Palo Alto University, Palo Alto, CA, United States

**Keywords:** internet interventions, maternal mental health, Mothers and Babies Course, postpartum depression, perinatal, prevention

## Abstract

Despite the availability of evidence-based postpartum depression (PPD) prevention and treatment interventions, perinatal persons continue to suffer. eHealth and mHealth tools to address mental health issues have grown exponentially, especially given the ubiquity of technology and the increased demand for telemental health resources. The Mothers and Babies Online Course (eMB), an 8-lesson prevention of PPD intervention, was digitally adapted to expand the reach of evidence-based interventions to perinatal persons with limited access to maternal mental health resources. This report describes the characteristics, behaviors, and feedback provided by users of the updated eMB website. Two hundred eight predominantly English-speaking U.S. residents enrolled in the eMB. Thirty-seven percent were either pregnant (*n* = 38) or postpartum (*n* = 39) women interested in learning skills to manage changes in their mood during and after pregnancy; 63% were health providers (*n* = 131) interested in learning how to support their patient communities. Seventy-six percent (*n* = 159) viewed at least one of the eight eMB lessons, with 50.9% exclusively viewing Lesson 1. Few (4.4%) viewed all eight lessons. The lessons were rated favorably on usefulness and understanding. Perinatal women engaged with interactive content at higher rates than health providers. Examining user behaviors and feedback is an essential developmental step before empirically testing the efficacy of digital tools. Future iterations of the eMB will incorporate these preliminary findings to provide perinatal persons with accessible web-based interventions that will hopefully reduce the incidence and negative consequences of postpartum depression.

## Introduction

Maternal mental health disorders impact the mood and functioning of perinatal persons, with approximately 15 to 20% of this population experiencing symptoms of perinatal mood and anxiety disorders (1). Postpartum depression (PPD), a condition affecting roughly 14.5% of new mothers, is among the most common maternal mental health disorders and the number one complication of birth (2, 3). If left untreated, mothers who experience PPD are at greater risk for maternal suicide and can experience other adverse outcomes concerning themselves and their babies (2–4). Screening for maternal mental health disorders has recently received more attention; however, perinatal depression continues to be undetected and undertreated (5).

The prevention and treatment of maternal mental health disorders require effective psychological interventions and pharmacological treatments. Effective approaches for preventing and treating PPD include talk therapies like cognitive behavioral therapy and interpersonal psychotherapy (1), which are effective in preventing and treating PPD (6). However, psychological interventions may not always be accessible to individuals who need them due to logistical and systemic issues, especially among ethnically and racially diverse perinatal persons who often suffer from inequalities in maternal healthcare and mental health access (7).

With technology becoming more accessible and highly utilized in our society, there has been an increase in internet- and mobile-based mental health interventions to address mental health disorders such as depression and anxiety (8). Internet-based interventions allow for standard therapy approaches (e.g., cognitive behavior therapy) to be effectively delivered and allow for the general population to access mental health services more easily with reduced barriers such as cost (9, 10), transportation and childcare (11), and social isolation, as was the case during the Covid-19 pandemic (12).

Many internet-based interventions are formatted to be convenient and accessible to individuals anytime and at their own pace (13). Online programs are frequently text-based and deliver psychoeducational information (14). For women who complete the full course of digital interventions, symptoms of depression and complicated grief have shown to be reduced (15). Still, barriers remain, such as low engagement for programs with limited human support (16). Similarly, recruitment for internet based PPD intervention programs can be challenging in reaching users it was designed for, or who would benefit from the intervention. Most recruitment in perinatal interventions comes from clinics, health providers, and self-referrals from online applications and media platforms (17). To further enhance online interventions and better understand the needs of organic users of perinatal interventions, we must examine their characteristics so recruitment and outreach efforts may be tailored with the goal of widespread global impact.

This study builds on a body of work using the Mothers and Babies Course [MB; (18)], a prevention of PPD intervention recognized by the U.S. Preventive Services Task Force as an evidence-based approach that should be recommended and made available to high-risk perinatal persons (19). The Mothers and Babies Online Course (eMB), a web-based adaptation of the face-to-face 8-lesson MB, was initially designed and pilot tested in a randomized controlled trial (RCT) among an international sample of English and Spanish-speaking pregnant persons (20). Although intervention effects were not demonstrated, pregnant persons with elevated depression symptoms who engaged with the eMB showed a reduced risk of PPD during the postpartum period. Follow-up participant feedback on the content, structure, and cultural relevance of the eMB revealed that perinatal persons preferred content that was tailored to their specific interests (e.g., stress management) and which addressed their unique maternal experiences (21). Furthermore, a desire for their partners and others to access the information in the eMB was a structural recommendation made to empower their support network about maternal mental health information. With the knowledge gained from the original RCT and subsequent participant feedback, the eMB was re-designed and re-launched as an online community resource for anyone interested in learning about maternal mental health. The purpose of this naturalistic study was to describe users of the newly designed eMB with an emphasis on perinatal persons and health providers. More specifically, we set out to examine a) who, among internet users, would be interested in the eMB?; b) what would be users' motivation for enrolling in the site?; and c) how would enrolled users access and rate the eMB? Using the information gathered from this study, the eMB can be modified to incorporate more information and resources tailored to the user's needs and serve as a reference for other online perinatal interventions.

## Materials and Methods

### Participants

Participants (*N* = 208) were 131 health providers (63%) and 77 perinatal women^1^ (50.6% were pregnant). The overall sample was mostly English-speakers (87.5%) residing in the U.S. (77.8%) who identified their ethnicity as non-Hispanic/non-Latinx (68.9%) and their race as predominantly White/European (69.8%). Health providers were older (*M* = 40.7 vs. *M* = 32.1, *p* < 0.001), mostly English-speaking (83.2 vs. 94.8%, *p* = 0.02) U.S. residents (72.0 vs. 87.7%, *p* < 0.001) who identified as non-Hispanic/non-Latinx (64.8 vs. 74.6%, *p* = 0.03). Among non-U.S. residents, health providers represented 14 countries (Argentina, Australia, Canada, Chile, China, Columbia, Costa Rica, India, Indonesia, Mexico, Panama, Paraguay, Spain, Turkey) while perinatal women residing outside of the U.S. were from Argentina, Canada, Spain, United Kingdom, and Uruguay. See Table 1 for a full description of all demographic characteristics.

**Table 1:** Participant demographic characteristics (*N* = 208).

	**Health providers (*n* = 131)**	**Perinatal persons (*n* = 77)**	***p***
Age (M, SD)	40.7 (10.2)	32.1 (5.2)	<0.001
	**Percent (%)**		
Female	99.0	100.0	*ns*
Woman	99.0	100.0	*ns*
Straight or mostly straight	88.9	94.5	*ns*
Ethnicity Non-Hispanic/Non Latinx	64.8	74.6	0.03
Race American Indian/Alaskan Native Asian Black/African American White/European Mixed race Other/not listed/prefer not to say	1.6 6.3 7.9 68.5 3.9 4.7	1.4 6.9 9.7 66.7 4.2 2.8	*ns*
Education University level Advanced degree	34.6 64.6	30.1 58.9	0.01
U.S. resident	72.0	87.7	<0.001
Maternal mental health knowledge			<0.001
A lot to a great deal	67.2	37.7	
Moderate amount	28.2	37.7	
A little to none	4.6	24.6	

### Measures

#### Eligibility Screening

Before consenting to participate, internet users indicated their age, sex, the reason for accessing the eMB and rated their subjective knowledge of maternal mental health. Being 18 years or older was the only inclusion criteria.

#### Baseline Questionnaire

Demographic information inquired about race, ethnicity, education, gender identity, sexual orientation, and country of residence. Perinatal history included current and past pregnancy and postpartum status. Participants were invited to indicate their current mood rating (i.e., *What is your mood like today? Think of your own life experiences to rate how you are feeling today*.) on a 1-question Quick Mood Scale which is a self-monitoring activity that is repeated as part of the original MB and the eMB content. Response choices ranged from 1 (worst mood) to 9 (best mood).

#### Mothers and Babies Online Course (eMB)

**Figure 1:**
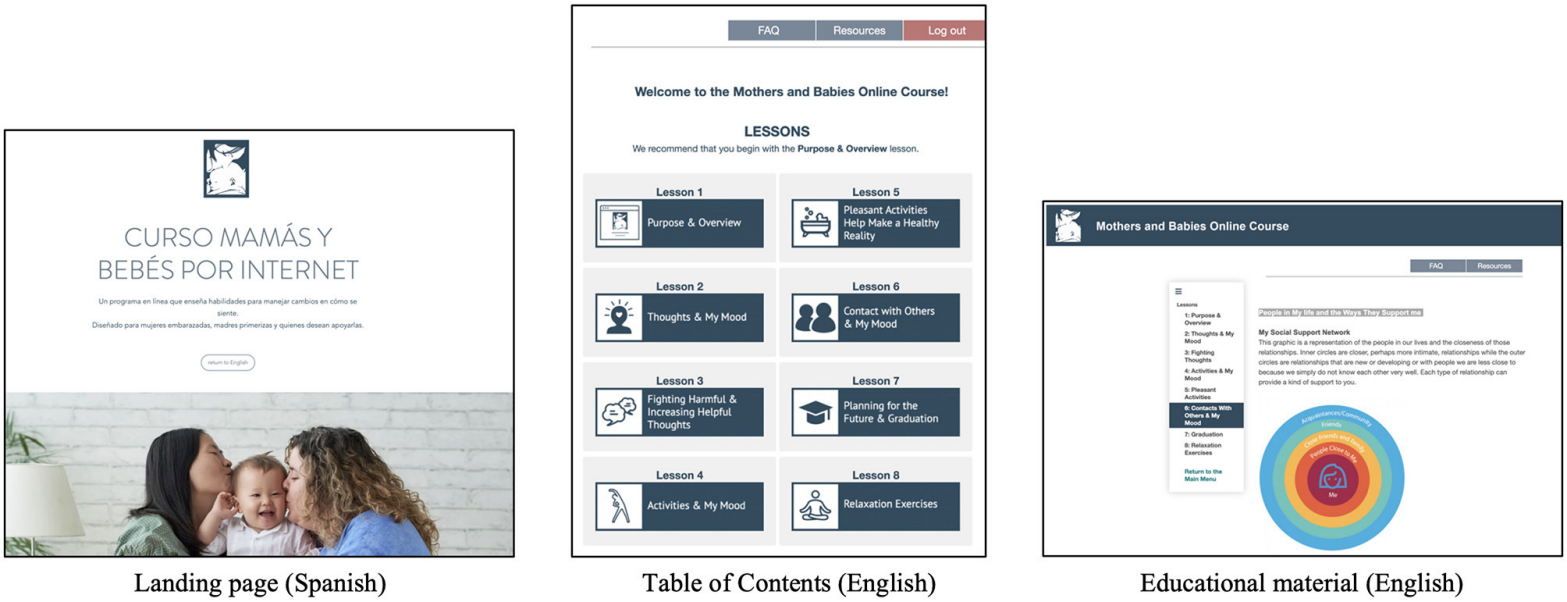
Sample images from the Mothers and Babies Online Course (eMB).

The eMB is a fully automated online prevention intervention based on the 8-lesson in-person group modality of the Mothers and Babies Course (22). The Mothers and Babies Course is based on cognitive-behavioral therapy (CBT) principles and integrates constructs from social learning theory and attachment theory to teach individuals how to manage changes in their mood during and after pregnancy. Given the online platform, the eMB was restructured into seven lessons that included videos, worksheets, and text pages to deliver the course content (See Figure 1). The first and seventh lessons introduce and conclude, respectively, the eMB. Lessons 2–6 emphasize core CBT constructs (i.e., cognitions, pleasant activities, social interactions) and their respective influence on perinatal mood changes. The eighth lesson houses pre-recorded audio relaxation and guided meditation exercises. The current version of the eMB allows participants to complete the lessons in their preferred order with minimal instructions or programing to influence participants. Participants were granted unlimited access to the full eMB and downloadable materials referenced in each lesson. The eMB was available in both English and Spanish.

Using a 1 (not at all) to 5 (very much) Likert scale, participants were invited to rate each lesson for usefulness (i.e., *How useful do you feel this lesson was*?) and understanding (i.e., *Was the information in this lesson easy to understand?*). The opportunity to provide qualitative feedback via a textbox was provided at the end of each lesson (i.e., *Please leave any questions, comments, or concerns regarding this lesson*).

#### Access and Engagement

For this report, the first lesson viewed, subsequent individual lessons viewed, and the number of views per lesson were used to assess participant access to the eMB. Twenty-one references to downloadable materials (via hyperlink) and thirty-nine fillable worksheets and content-related questions were integrated into Lessons 1 through 7 to support the application of key eMB constructs. The number and type of downloadable resources were specific to each lesson's core CBT construct and included, at minimum, a lesson-specific Quick Mood Scale. Participant engagement was measured via the number of clicks on downloadable materials, content-related questions answered, and the number of worksheets that were initiated or completed.

#### Procedures

Participants were recruited through email invitations, social media postings, and word-of-mouth. Recruitment materials invited individuals to participate if they had an interest in learning skills to manage changes in their feelings during and after pregnancy. Pregnant people, their partners, healthcare providers, and other members of their support system were specified as those who could participate in the study. Both individuals and organizations who regularly contact perinatal persons were targeted for all recruitment methods. Additionally, the study website (www.emb.health), was organically available to internet users via search engines. The study website described participation details and directed users to the intervention site where they were screened for eligibility and redirected to the login page for immediate access to the eMB lessons. The English-language eMB was launched in March 2020 and the Spanish-language version in March 2021. Data collected up until August 2021 on both sites are described in this report. The Institutional Review Board at Palo Alto University approved all procedures.

## Results

**Figure 2:**
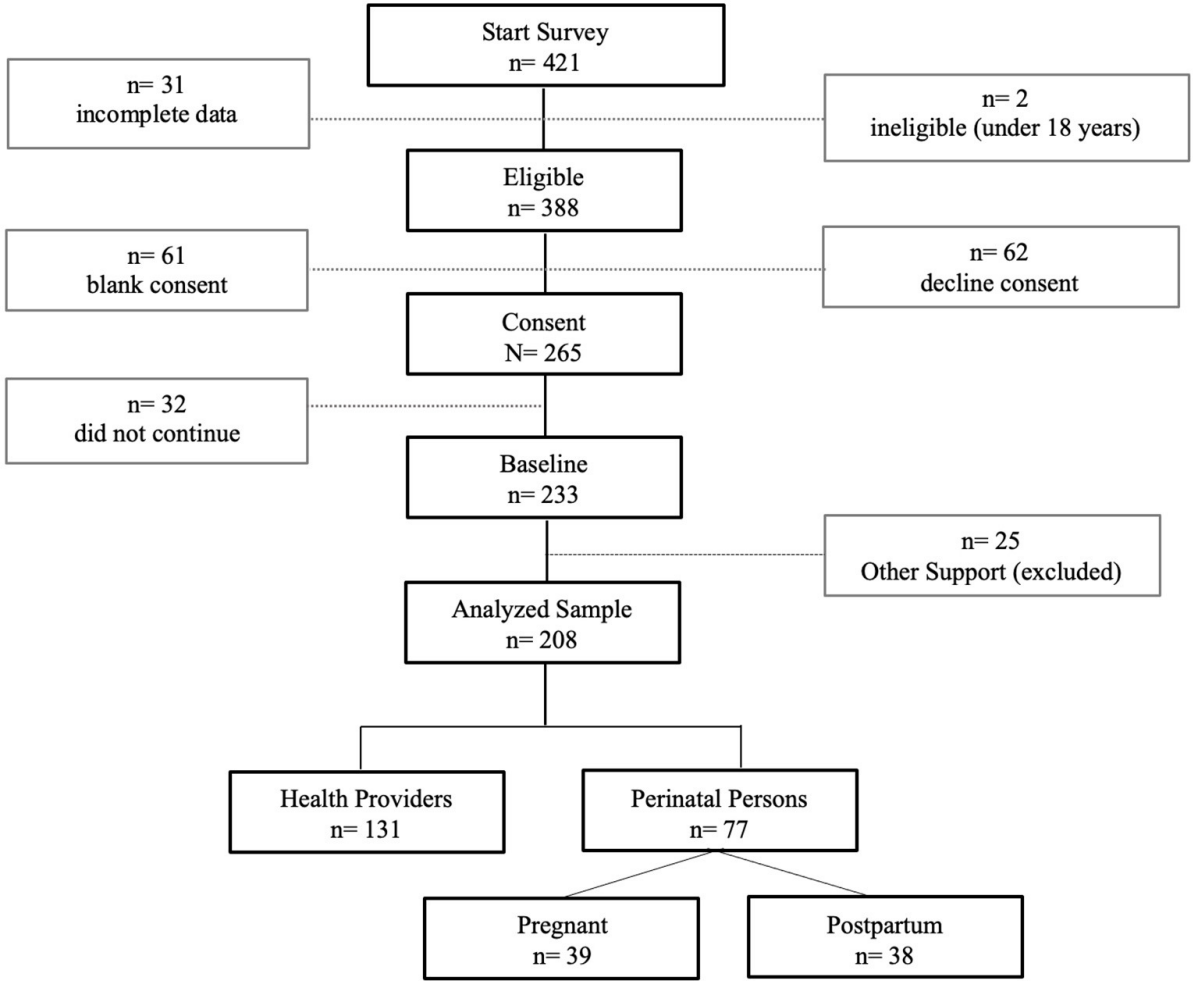
Participant recruitment and enrollment.

Four hundred and twenty-one internet users visited the study website (see Figure 2). Of these, 31 immediately exited the site or had insufficient data to determine eligibility; two internet users were under 18 years of age (i.e., ineligible). Out of the remaining 388 eligible participants, 233 consented to participate and continued to the baseline questionnaire. Participants were asked to indicate why they were interested in accessing the eMB (e.g., for myself, to support my partner, etc.) and were categorized into three primary groups - perinatal persons, health providers, and other support (e.g., partners, researchers). Those who identified as other support (*n* = 25) were excluded from further analysis given the variability of their motivation to enroll in the eMB. For example, only two participants indicated that they were either a partner or a family member wanting to support a perinatal person. While the remaining 23 described their motivation in non-specific terms (e.g., “Testing site for use with our program”). The final sample were 208 internet users who identified as health providers (*n* =131) and individuals who were pregnant (*n* = 38) or within 1 year postpartum (*n* = 38).

### Perinatal Characteristics

Most participants (86.5%) indicated either a current or past pregnancy; among health providers, 78.6% were mothers or had previously given birth (Table 2). The number of births differed between the health providers and perinatal women such that the latter was either pregnant with their first child (39.5%) or reported 1–2 prior births (57.9%). In contrast, very few health providers had not given birth (2%) and, among those who had, a majority had done so 1–2 (81.2%) or 3–4 (15.9%) times prior.

**Table 2:** Participant perinatal characteristics (*N* = 208).

	**Health providers (*n* = 131)**	**Perinatal persons (*n* = 77)**	***p***
Yes, ever pregnant	78.6	100	<0.001
Number of previous births			<0.001
None	2.0	39.5	
1-2	81.2	57.9	
3-4	15.9	1.3	
5+	1.0	1.3	
Pregnancy status			
Currently pregnant	–	50.6	
Weeks pregnant (M, SD)	–	23.4	
Gave birth in last year	–	(11.2)	
Gave birth over a year ago	73.3	49.4	

### Quick Mood Scale Rating and Knowledge About Maternal Mental Health

The current mood rating for the total sample was 6.26 (*SD* = 1.56; range = 1–9) with pregnant and postpartum women (*M* = 5.66, *SD* = 1.24, and *M* = 5.74, *SD* = 1.97, respectively) endorsing a lower mood than health providers (*M* = 6.58, *SD* = 1.42; *F*(2.197) = 7.99, *p* < 0.001). Overall, participants indicated higher knowledge about maternal mental health (88%). However, relative to perinatal women, health providers rated their knowledge at the highest levels (37.7 vs. 67.2%, respectively), whereas perinatal women believed their knowledge about maternal mental health was minimal to none (24.6 vs. 4.6%, respectively; *X*^2^(4) = 25.44, *p* < 0.001).

### eMB Access

Of the 208 participants, 47 (22.6%) never accessed the intervention site, and two (1%) logged in but exited without viewing any of the eMB lessons. Group comparisons did not reveal any significant differences in demographic characteristics between those who did and did not view the eMB lessons. However, health providers bypassed logging in and viewing eMB lessons at a higher rate than perinatal women (25.2 vs. 18.2%, respectively). Within the perinatal sample, a higher proportion of pregnant women bypassed logging in and viewing eMB lessons than postpartum women (28.2 vs. 7.9%, respectively; *p* = 0.02).

Of the 159 (76.4%) participants who viewed at least one lesson, 90.6% selected Lesson 1 as the initial lesson to view. All eight lessons were viewed at least once with a sample average of two individual lessons viewed (*M* = 2.14, *SD* = 1.78). Lessons 1 and 2 accounted for the most unique views (50.9 and 25.8%, respectively), and Lesson 6 accounted for the fewest unique views (0.6%). Five participants, each, viewed all eight lessons or at least four lessons. Participants were able to revisit lessons based on their preferences. As such, repeated views were highest for Lesson 1 (range = 2–6) and Lessons 4 and 5 (range = 2–4). Cumulative lesson views per participant, regardless of the lessons selected, ranged from 1–15, with most participants viewing each lesson once (43.4%), twice (23.9%), or three (10.7%) times. Among perinatal participants, more postpartum women viewed the eMB lessons than pregnant women (92.1 vs. 71.8%, respectively; *X*^2^(2) = 5.90, *p* = 0.05). No other meaningful differences emerged in how participants accessed the eMB lessons.

### eMB Usefulness and Understanding Ratings

Sixty-four participants (40.3%) who viewed eMB content provided feedback on at least one lesson. As expected, Lesson 1 was evaluated by the largest number of participants (*n* =57) and Lesson 8 by the fewest (*n* = 3), which was consistent with the frequency of viewing individual lessons. Usefulness and understanding ratings for all lessons ranged from 3 (neutral) to 5 (very much), except for Lesson 1, which received 13 (23.6%) usefulness ratings of 1 (not at all) or 2 (a little), mostly from perinatal women. The remaining lessons received the highest usefulness rating of 5 (very much) by at least 50% of participants and up to 70% of participants who rated it (Lesson 4 and Lesson 7, respectively). Similarly, the level of understanding was in the “very much” range by at least 70.2% (Lesson 2) and up to 100% of participants (Lessons 4 through 8). Group comparisons revealed that health providers rated the usefulness and understanding of Lessons 1 and 2 more favorably than perinatal women. The opposite was true for perinatal women's ratings of Lessons 3, 4, 6, and 7. Both groups rated the Lesson 5 content equally on usefulness and understanding, while Lesson 8 was only rated by health providers.

Few qualitative comments were received at the end of each lesson. However, the feedback was generally positive (e.g., “*It was helpful to identify activities to do with the baby. The transition to motherhood seems really limiting and this was a helpful exercise*.”), a reflection to learning new techniques or skills (e.g., “*I felt learning how to reframe negative thoughts in writing was very helpful!*”), and constructive (e.g., “*Is lesson 2.2 introduced here to be continued in the next lesson, I felt I needed further elaboration on taking back harmful thoughts. It was left on a negative note*.”).

### eMB Engagement in Interactive Content

Thirty-nine fillable worksheets and content-related questions were included in Lessons 2–7. The worksheets and questions were designed to help participants consider the eMB lesson constructs within the context of their own perinatal experience. In total, 26% (*n* = 43) of participants who viewed eMB lessons responded to at least one worksheet or content-related question. Perinatal women responded to worksheets and content-related questions at a higher rate (33.3%) than health providers (22.9%) with pregnant women responding to more worksheets and content-related questions than postpartum women (*M* = 13.8, *SD* = 10.4 vs. *M* = 8.4, *SD* = 8.6, respectively). No significant group differences were found between those who typed information into the worksheets and content-related questions and those who did not.

Downloadable resources accessible by clicking on hyperlinks embedded in each lesson were an additional metric to assess participant engagement. In total, twenty-one downloadable resource hyperlinks were integrated into Lessons 1–7. Half (49.1%, *n* = 78) of the participants who viewed the eMB lessons clicked on at least one downloadable resource, with a majority (74.4%, *n* = 54) selecting to view downloadable resources 1–2 times and up to 10 times. Over half of the perinatal women (54%, *n* = 34) clicked on the downloadable resources (vs. 45.8% of health providers, *n* = 44), with slightly more postpartum women accessing the downloadable resources than pregnant women (54.3 vs. 53.6%, *ns*).

## Discussion

Interventions for postpartum depression have received more attention due to increased maternal mortality rates and the impact of postpartum depression on birthing people, their children, and their family members (23). Technology-based postpartum depression interventions have addressed some of the barriers (e.g., stigma, lack of access and knowledge) perinatal persons face, which has begun to improve mental health outcomes (23). To date, some of these interventions have included online programs and telephone-based support, which have been shown to reduce postpartum depression symptoms (24).

This study set out to examine the newly designed Mothers and Babies Online Course (eMB). Armed with the lessons learned from an earlier trial [i.e., (19, 20)], this version of the eMB opened access to non-perinatal persons and updated the presentation and delivery of the eMB content to include images and references that represented intersectional identities, included more interactive activities and resources, and added brief videos and pre-recorded audio files. As a preliminary step, the primary aim described in this report focused on identifying who and why users enrolled, how they accessed the intervention content when given unlimited access and limited restrictions and obtained feedback to improve the eMB experience.

An unexpected finding from this study was the greater proportion of non-perinatal persons enrolled. Recruitment and outreach efforts targeted perinatal platforms, listservs, and providers serving these communities. However, most of those who enrolled in the eMB were health providers and other support individuals (e.g., researchers). Recruitment began, coincidently, with the onset of the Covid-19 pandemic and subsequent shelter-in-place mandates. Health providers were expected to shift how they delivered their services, including what they recommended to their patient communities, given limited availability and access to in-person services. Our team has been researching and disseminating digital interventions for PPD for over 15 years, and we were optimistic that the online delivery and minimal onboarding criteria would address the urgent need for available perinatal mental health resources. Most likely, however, health providers were overwhelmed by the increased demand for mental health services that could be delivered digitally or via telehealth. Further, the pandemic brought perinatal mental health to the forefront and highlighted the disproportionate occurrence of adverse birth outcomes and mental distress among perinatal persons, especially those from diverse socioeconomic and cultural backgrounds (25). As such, health providers and other support persons may have enrolled in the site intending to share it but lacked the bandwidth to do so, given pandemic-related stressors. Alternatively, it is possible that health providers who enrolled were seeking additional resources to increase their knowledge and develop their competencies working with perinatal populations [e.g., (26)] given that the reproductive health and associated mental health issues remains a specialized area of training in many health fields that intersect with maternal healthcare.

Despite efforts to open the eMB to perinatal people and their non-perinatal support networks, approximately half of those who visited the intervention site did not enroll and among those who did, many minimally engaged with the eMB content. Retention, attrition, and engagement issues are well-established challenges of online interventions (27). Regardless of the recruitment outcomes, perinatal women who enrolled in the study were exposed to evidence-based and theory driven educational content, skills, and techniques to reduce postpartum depression risk (19). Perinatal women engaged the most with the eMB as they recorded the most lesson views, accessed the most downloadable materials, and responded to worksheets and content-related questions at a higher rate than enrolled health providers. Further, pregnant participants reported the lowest current mood, followed by postpartum women. Health providers' lower engagement was potentially due to a reduced need to learn about perinatal mental health given their expressed higher level of knowledge about the topic during the study enrollment. The aims of this study did not include measuring the efficacy of the eMB against depression symptoms. As such, changes in depression mood ratings were not examined in this report. However, it has been well established that women in the U.S., especially pregnant women, go undiagnosed and, consequently, untreated. Furthermore, even when treatment is received, their needs often are not fully met (5). To improve on engagement and to reach perinatal women with a greater need for the type of information delivered by the eMB, future efforts will use indicated and selective approaches to recruitment (i.e., target depressed or high-risk perinatal persons) instead of the universal approach used in this study.

This study successfully addressed the aim of examining how enrolled participants accessed and engaged with the eMB. In the previous eMB (20), participants were restricted to viewing the lessons in a linear and chronological order. In contrast, the current version of the eMB did not provide any restrictions and only recommended that participants begin with Lesson 1. Regardless, in both studies, participants almost exclusively viewed Lesson 1. This replicated finding suggests that the initially viewed lesson may be the most important for engaging and retaining participants and delivering core psychological constructs that will lead to the most significant positive outcomes, especially in a fully automated internet intervention such as the eMB (28). Findings from this study also suggest that the added flexibility of digital interventions (i.e., users can choose the order and quantity of content viewed) may not be necessary for all potential users and that when given the option, users will likely continue to view materials in a systematic order as is done in traditional, face-to-face psychological interventions. Unlike traditional, face-to-face psychotherapy, however, online interventions may lack the structure to influence whether a user returns to the site (e.g., social desirability). As such, future iterations of the eMB would benefit from clearly outlining and delivering core constructs early in the intervention design and structure each lesson so that it can be viewed independently from other lessons. Incorporating downloadable materials and interactive worksheets allowed participants to stay engaged with the eMB when not logged in to the website. Though this study did not record the duration of time spent completing the lessons or viewing downloadable materials, future iterations will incorporate this function to better measure engagement among perinatal persons.

Lesson-specific content ratings and feedback on usefulness and understanding suggested that our efforts to redesign the presentation and delivery of eMB content was successful. An interesting caveat to this finding was perinatal women's lower usefulness ratings of Lesson 1 relative to the remaining lessons and those submitted by health providers. In the original in-person Mothers and Babies Course, this initial lesson is foundational and provides the theoretical justification for the intervention's cognitive-behavioral therapy approach. Against the backdrop of their self-reported limited knowledge about maternal mental health, it makes sense that perinatal women rated this lesson less favorably as it did not deliver specific skills or techniques to reduce changes in mood. Future iterations of the eMB will need to address this as it may have subsequent implications on retention and engagement which will impact future efforts to examine the efficacy of the eMB to prevent PPD.

This report is not without a few limitations that are worth noting. Future iterations of the eMB will need to incorporate targeted recruitment efforts and increase engagement (29), especially if the goal is to demonstrate that it is effective at preventing PPD. Related, the applied engagement metrics are likely underestimates of actual engagement given that we did not track duration of use and were unable to measure if participants viewed or listened to pre-recorded content, aspects of the intervention that were added in response to feedback received in the previous version of the eMB. As such, additional structural metrics will need to be implemented to accurately measure how users engage with the online intervention and which aspects may play a key role in the management of perinatal mood changes.

In sum, pregnant and postpartum persons are interested in digital interventions to prevent PPD (30). This study also demonstrated a high interest in digital interventions, especially among health providers and other support individuals working with perinatal persons. As the availability of online interventions continues to grow and become more widely acceptable, user preferences, behaviors, and feedback during the design phase will be essential to incorporate before examining their efficacy for treating mental health disorders. Health providers, partners, and family members of perinatal persons may also benefit from accessing these interventions as they too may rely on these resources to support perinatal individuals, given that the mental health of perinatal persons remains a specialized expertise and not a standard consideration of maternal healthcare (31).

## Data Availability Statement

The datasets presented in this article are not readily available due to participants' data privacy. Requests to access the datasets should be directed to AB, abarrera@paloaltou.edu.

## Ethics Statement

The studies involving human participants were reviewed and approved by Institutional Review Board, Palo Alto University. Written informed consent for participation was not required for this study in accordance with the national legislation and the institutional requirements.

## Author Contributions

AB was responsible for overseeing all aspects of this manuscript, including the initial study design, aims, analyses, and structure. SM and AR drafted initial versions of the Introduction and Materials and Methods, Results sections, conducted preliminary analyses. SM was responsible for data organization and AR was responsible for managing citations. All authors contributed to editing the final version of this manuscript and completion of this article.

## Conflict of Interest

The authors declare that the research was conducted in the absence of any commercial or financial relationships that could be construed as a potential conflict of interest.

## Publisher's Note

All claims expressed in this article are solely those of the authors and do not necessarily represent those of their affiliated organizations, or those of the publisher, the editors and the reviewers. Any product that may be evaluated in this article, or claim that may be made by its manufacturer, is not guaranteed or endorsed by the publisher.

## Footnotes

^1^All participants identified their gender as woman. As such, they will be referred to as women/woman in this report (e.g., perinatal women, pregnant woman) instead of perinatal/birthing persons which is more gender inclusive.
